# Editorial: Anti-inflammatory diet in autoimmune diseases

**DOI:** 10.3389/fnut.2024.1497058

**Published:** 2024-10-22

**Authors:** Ifigenia Kostoglou-Athanassiou, Panagiotis Athanassiou

**Affiliations:** ^1^Department of Endocrinology, Diabetes and Metabolism, Asclepeion Hospital, Athens, Greece; ^2^Department of Rheumatology, St. Paul's Hospital, Thessaloniki, Greece

**Keywords:** anti-inflammatory, diet, autoimmune, autoimmunity, curcumin

Nutrition seems to play a role in the prevention or amelioration of autoimmunity (1). Autoimmunity leads to inflammation (2, 3). Nutritional factors which may act as anti-inflammatory agents, like antioxidants and many vitamins may prevent or ameliorate autoimmune diseases (4). This has been studied extensively in the context of rheumatoid arthritis (RA) (4). It has also been studied extensively in the context of inflammatory bowel disease (5). Dietary factors may prevent or ameliorate rheumatoid arthritis (6). In this Research Topic we aimed to study the effect of dietary factors which may have anti-inflammatory properties in the prevention and management of autoimmune diseases.

Curcumin is a product of the root of the plant Curcuma longa (7). It has been used over the centuries in many Asian countries as a spice and as a traditional medicine. Recent studies have confirmed that curcumin does have beneficial effects in the prevention and treatment of autoimmune diseases, including, RA and inflammatory bowel disease (8). Curcumin is widely used by patients for its beneficial health effects, including anti-inflammatory and cancer preventive effects. In their study Kroon et al. investigated the levels of curcumin and its various metabolites in a group of patients who took curcumin. They also investigated the effect of various agents taken by the patients to improve curcumin levels and its beneficial effects such as piperine. Plasma samples were studied with and without pretreatment with β-glucuronidase in order to measure conjugated and unconjugated curcumin levels. In their study they found that the addition of β-glucuronidase in the plasma samples increased curcumin levels. They found that the use of curcumin supplements may not increase curcumin levels to therapeutic standards. They cautioned medical practitioners to be aware of the fact that the mere use of curcumin supplements may not lead to therapeutic levels of the substance within the organism.

Saturated fatty acids are implicated in the pathogenesis of various chronic systemic diseases such as atherosclerosis. Saturated fatty acids may also play a role in the pathogenesis of autoimmune diseases such as RA (9). Yao et al. investigated the potential causal relationship between saturated fatty acids and RA using Mendelian randomization analysis. Genome wide association data for RA and elevated saturated fatty acids were obtained from an open database of genetic data. The authors performed Mendelian randomization analysis of the causal effect of elevated saturated fatty acids on RA occurrence. They observed a positive link between saturated fatty acids and the risk of RA. They further evaluated their findings performing sensitivity analysis. In order to further confirm their observations they performed reverse Mendelian randomization analysis and they did not observe any causal effect of RA on the risk for elevated saturated fatty acids. Consequently, this study provided preliminary data on a possible etiological relationship between saturated fatty acids and the risk of RA. This study did not look at the mechanisms involved in the relationship between saturated fatty acids and a systemic autoimmune disease like RA. However, it has been shown that a diet high in saturated fatty acids may promote T lymphocyte activation and T lymphocyte differentiation toward Th1 and Th17 subtypes (10). Additionally, saturated fatty acids acting within the intestine may promote the production of inflammatory cytokines (11). The authors, Yao et al., admit within their discussion that the method they used cannot identify the exact biological mechanisms, which may be implicated in the relationship between elevated saturated fatty acids and the risk of RA. The authors also admitted that there is a lack of data in the open genetic database they used on the specific subtypes of saturated fatty acids which may be involved in the risk of RA. Consequently, this study does not provide any evidence on the specific subtypes of saturated fatty acids which should be eliminated from the diet to reduce the risk of RA. Thus, the findings of Yao et al. suggest that elevated saturated fatty acids may increase the risk for the development of RA.

Synovial involvement and inflammation is a characteristic of RA (12). Lu et al. investigated in an epidemiological study, utilizing data from the NHANES (NATIONAL HEALTH AND NUTRITIOIN EXAMINATION SURVEY) cohort over a period spanning from 1999 to 2018, the relationship between the dietary inflammatory potential and immune inflammatory markers. Their investigation involved a population of 2,500 RA patients. The dietary inflammatory potential was calculated by the dietary inflammatory index score based on dietary recall interviews. They found a positive correlation between the dietary inflammatory potential and immune inflammatory markers in the cohort of RA patients they studied.

The relationship between polyunsaturated fatty acids and autoimmune rheumatic diseases remains controversial. Xu et al. investigated the relationship between polyunsaturated fatty acids and autoimmune rheumatic diseases using Mendelian randomization analysis. Their findings indicated that an increased genetic predisposition for elevated levels of eicosapentaenoic acid (EPA) may be related to a decreased susceptibility to psoriatic arthritis, suggesting that dietary supplementation with EPA may decrease the risk of psoriatic arthritis onset.

In conclusion, it appears that diet via various nutritional factors may play a role in the pathogenesis, prevention and management of systemic autoimmune diseases.
